# Evolution of phototaxis

**DOI:** 10.1098/rstb.2009.0072

**Published:** 2009-10-12

**Authors:** Gáspár Jékely

**Affiliations:** Max Planck Institute for Developmental Biology, Spemannstrasse 35, 72076 Tübingen, Germany

**Keywords:** phototaxis, evolution, eukaryote, convergent evolution, stigma, rhodopsin

## Abstract

Phototaxis in the broadest sense means positive or negative displacement along a light gradient or vector. Prokaryotes most often use a biased random walk strategy, employing type I sensory rhodopsin photoreceptors and two-component signalling to regulate flagellar reversal. This strategy only allows phototaxis along steep light gradients, as found in microbial mats or sediments. Some filamentous cyanobacteria evolved the ability to steer towards a light vector. Even these cyanobacteria, however, can only navigate in two dimensions, gliding on a surface. In contrast, eukaryotes evolved the capacity to follow a light vector in three dimensions in open water. This strategy requires a polarized organism with a stable form, helical swimming with cilia and a shading or focusing body adjacent to a light sensor to allow for discrimination of light direction. Such arrangement and the ability of three-dimensional phototactic navigation evolved at least eight times independently in eukaryotes. The origin of three-dimensional phototaxis often followed a transition from a benthic to a pelagic lifestyle and the acquisition of chloroplasts either via primary or secondary endosymbiosis. Based on our understanding of the mechanism of phototaxis in single-celled eukaryotes and animal larvae, it is possible to define a series of elementary evolutionary steps, each of potential selective advantage, which can lead to pelagic phototactic navigation. We can conclude that it is relatively easy to evolve phototaxis once cell polarity, ciliary swimming and a stable cell shape are present.

## 1. Phototaxis in prokaryotes

Most prokaryotes are unable to sense the direction of light, because at a small scale it is very difficult to make a detector that can distinguish a single light direction. Still, prokaryotes can measure light intensity and move in a light-intensity gradient. Some gliding filamentous prokaryotes can even sense light direction and make directed turns, but their phototactic movement is very slow. Some species among both eubacteria and archaebacteria (archaea) are phototactic (Scharf & Wolff 1994; Armitage & Hellingwerf 2003). In most cases the mechanism of phototaxis is a biased random walk, analogous to bacterial chemotaxis. Halophilic archaebacteria, such as *Halobacterium salinarum*, use sensory rhodopsins (SRs) for phototaxis (Luecke *et al.* 2001; Spudich 2006). Rhodopsins are 7-transmembrane proteins that bind retinal as a chromophore. Light triggers the all-*trans*/13-*cis* isomerization of retinal (Yan *et al.* 1990), which leads to phototransductory signalling via a two-component phosphotransfer relay system. *Halobacterium salinarum* has two SRs, SRI and SRII, which signal via the transducer proteins HtrI and HtrII (halobacterial transducers for SRs I and II), respectively (Gordeliy *et al.* 2002; Sasaki & Spudich 2008). The downstream signalling in phototactic archaebacteria involves CheA, a histidine kinase, which phosphorylates the response regulator, CheY (Rudolph & Oesterhelt 1995). Phosphorylated CheY induces swimming reversals. The two SRs in *Halobacterium* have different functions. SRI acts as an attractant receptor for orange light and, through a two-photon reaction, a repellent receptor for near-UV light, while SRII is a repellent receptor for blue light. Depending on which receptor is expressed, if a cell swims up or down a steep light gradient, the probability of flagellar switch will be low. If light intensity is constant or changes in the wrong direction, a switch in the direction of flagellar rotation will reorient the cell in a new, random direction (McCain *et al.* 1987). As the length of the tracks is longer when the cell follows a light gradient, cells will eventually get closer to or further away from the light source. This strategy does not allow orientation along the light vector and only works if a steep light gradient is present (i.e. not in open water).

Some cyanobacteria (e.g. *Anabaena*, *Synechocystis*) can slowly orient along a light vector. This orientation occurs in filaments or colonies, but only on surfaces and not in suspension (Nultsch *et al.* 1979; Choi *et al.* 1999). The filamentous cyanobacterium *Synechocystis* is capable of both positive and negative two-dimensional phototactic orientation. The positive response is probably mediated by a bacteriophytochrome photoreceptor, TaxD1. This protein has two chromophore-binding GAF domains, which bind biliverdin chromophore (Bhoo *et al.* 2001), and a C-terminal domain typical for bacterial taxis receptors (MCP signal domain). TaxD1 also has two N-terminal transmembrane segments that anchor the protein to the membrane. (Zhulin 2000; Bhaya 2004; Yoshihara & Ikeuchi 2004). The photoreceptor and signalling domains are cytoplasmic and signal via a CheA/CheY-type signal transduction system to regulate motility by type IV pili (Yoshihara *et al.* 2000). TaxD1 is localized at the poles of the rod-shaped cells of *Synechococcus elongatus*, similarly to MCP containing chemosensory receptors in eu- and archaebacteria (Gestwicki *et al.* 2000). How the steering of the filaments is achieved is not known. The slow steering of these cyanobacterial filaments is the only light-direction sensing behaviour prokaryotes could evolve owing to the difficulty in detecting light direction at this small scale.

## 2. Phototaxis in eukaryotes

Eukaryotes evolved for the first time in the history of life the ability to follow light direction in three dimensions in open water. The strategy of eukaryotic sensory integration, sensory processing and the speed and mechanics of tactic responses is fundamentally different from that found in prokaryotes (Häder & Jori 2001). Both single-celled and multi-cellular eukaryotic phototactic organisms have a fixed shape, are polarized, swim in a spiral and use cilia for swimming and phototactic steering. Signalling can happen via direct light-triggered ion currents, adenylyl cyclases or trimeric G-proteins. The photoreceptors used can also be very different (see below). However, signalling in all cases eventually modifies the beating activity of cilia. The mechanics of phototactic orientation is analogous in all eukaryotes. A photosensor with a restricted view angle rotates to scan the space and signals periodically to the cilia to alter their beating, which will change the direction of the helical swimming trajectory.

**Figure 1.**
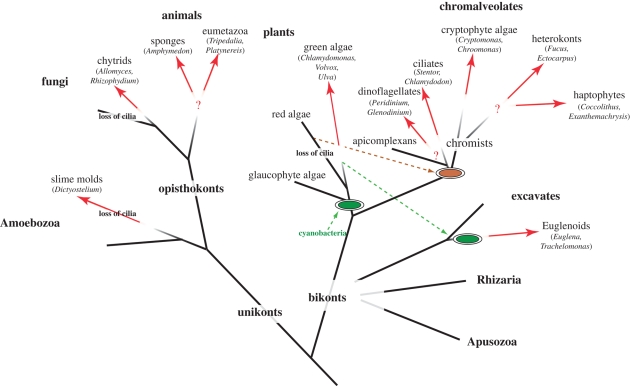
The distribution of three-dimensional phototaxis in the tree of eukaryotes. Red arrows indicate the likely point of origin of phototaxis in a given group. Question marks indicate uncertainties regarding independent or common origin.

Below I discuss the diversity of photopigments and morphological solutions that are used to achieve phototactic orientation in diverse eukaryotes. Three-dimensional phototaxis can be found in five out of the six eukaryotic major groups (opisthokonts, Amoebozoa, plants, chromalveolates, excavates, rhizaria). For an overview of eukaryote diversity, phylogeny, taxonomy and the rooting of the eukaryote tree, see Stechmann & Cavalier-Smith (2002), Cavalier-Smith (2003, 2004), Simpson & Roger (2004), Adl *et al.* (2005), Keeling *et al.* (2005) and Baldauf (2008) (figure 1).

## 3. Plants

Plants originated via a primary endosymbiotic event between a biciliate protozoan host and a cyanobacterium, the ancestor of chloroplasts. Following the origin of chloroplasts, plants diverged into three lineages, glaucophyte algae (Glaucophyta), red algae (Rhodophyta) and green algae+land plants (Viridaeplantae). Of the three lineages, pelagic phototaxis is only present in green plants.

Glaucophytes are a small group of freshwater algae. They lack stigmata and phototaxis. A photophobic reaction, as described in *Cyanophora paradoxa* (Häder 1985), can help glaucophytes to avoid bright light.

Red algae lack cilia in all stages of their life cycle and consequently lack the ability of helical swimming. Consistent with this, three-dimensional phototaxis and stigmata are absent from the whole group. Red algae find optimal light conditions using surface gliding and two-dimensional phototaxis, as described in *Porphyridium cruentum* (Nultsch & Schuchart 1980).

In green algae, pelagic three-dimensional phototaxis is very widespread and several species, both unicellular and multi-cellular, harbour conspicuous stigmata (singular, stigma, also called eyespots; Halldal 1958). For a comprehensive list of phototactic green algae, see Bendix (1960).

**Figure 2.**
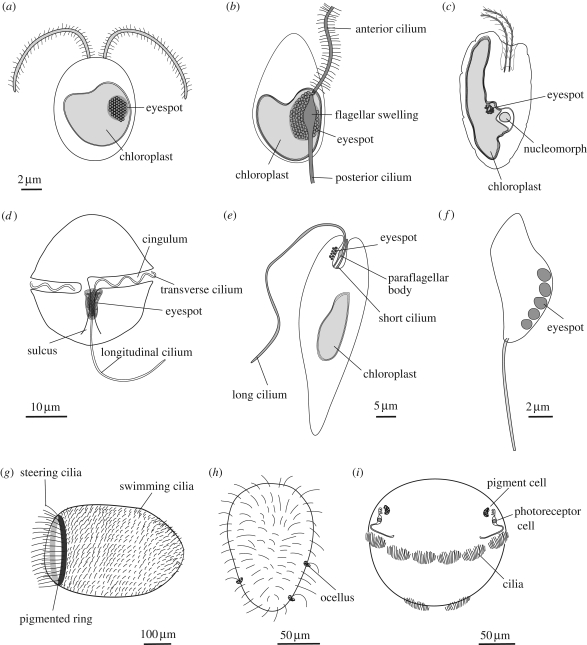
The diversity of phototactic eukaryotes (*a*) a green alga (scale bar, 2 µm), (*b*) a heterokont zoospore, (*c*) a cryptomonad alga, (*d*) a dinoflagellate (scale bar, 10 µm), (*e*) *Euglena* (scale bar, 5 µm), (*f*) a chytrid zoospore (scale bar, 2 µm), (*g*) a sponge larva (scale bar, 100 µm), (*h*) a cnidarian larva (scale bar, 50 µm) and (*i*) a polychaete larva (scale bar, 50 µm).

Green algae have a stigma located in the outermost portion of the chloroplast, directly underneath the two chloroplast membranes (figure 2). The stigma is made of tens to several hundreds of lipid globules, which often form hexagonal arrays and can be arranged in one or more rows. The lipid globules contain a complex mixture of carotenoid pigments, which provide the screening function and the orange-red colour (Grung *et al.* 1994), as well as proteins that stabilize the globules (Renninger *et al.* 2001). The stigma is located laterally, in a fixed plane relative to the cilia, but not directly adjacent to the basal bodies (Arnott & Brown 1967; Melkonian & Robenek 1979). The fixed position is ensured by the attachment of the chloroplast to one of the ciliary roots (Melkonian 1978). The pigmented stigma is not to be confused with the photoreceptor. The stigma only provides directional shading for the adjacent membrane-inserted photoreceptors (the term ‘eyespot’ is therefore misleading). Stigmata can also reflect and focus light like a concave mirror, thereby enhancing sensitivity.

In the best-studied green alga, *Chlamydomonas reinhardtii*, phototaxis is mediated by a rhodopsin pigment, as first demonstrated by the restoration of normal photobehaviour in a blind mutant by analogues of the retinal chromophore (Foster *et al.* 1984). Two archaebacterial-type rhodopsins, *Chlamydomonas* sensory rhodopsin A and B (CSRA, CSRB), also called channelrhodopsin-1 and -2 (Nagel *et al.* 2002, 2003), were identified as phototaxis receptors in *Chlamydomonas* (Sineshchekov *et al.* 2002). Both proteins have an N-terminal 7-transmembrane portion, similar to archaebacterial rhodopsins, followed by an approximately 400 residue C-terminal membrane-associated portion. CSRA and CSRB act as light-gated cation channels and trigger depolarizing photocurrents (Sineshchekov *et al.* 2002; Berthold *et al.* 2008). CSRA was shown to localize to the stigma region using immunofluorescence analysis (Suzuki *et al.* 2003). Individual RNAi depletion of both CSRA and CSRB modified the light-induced currents and revealed that CSRA mediates a fast, high-saturating current while CSRB a slow, low-saturating one. Both currents are able to trigger photophobic responses and can have a role in phototaxis (Govorunova *et al.* 2004; Berthold *et al.* 2008), although the exact contribution of the two receptors is not yet clear.

Other green algae, including *Haematococcus* (Litvin *et al.* 1978), *Spermatozopsis* (Kreimer *et al.* 1991) and *Volvox*, have similar photoelectric cascades and probably use similar type-I SRs for phototaxis (Sineshchekov & Spudich 2005).

As in all bikonts (plants, chromalveolates, excavates, rhizaria), green algae have two cilia, which are not identical. The anterior cilium is always younger than the posterior one (Cavalier-Smith 2002*b*, 2009). In every cell cycle, one daughter cell receives the anterior cilium and transforms it into a posterior one. The other daughter inherits the posterior, mature cilium. Both daughters then grow a new anterior cilium.

As all other ciliary swimmers, green algae always swim in a spiral. The handedness of the spiral is robust and is guaranteed by the chirality of the cilia. The two cilia of green algae have different beat patterns and functions. In *Chlamydomonas*, the phototransduction cascade alters the stroke pattern and beating speed of the two cilia differentially in a complex pattern (Josef *et al.* 2005, 2006). This results in the reorientation of the helical swimming trajectory as long as the helical swimming axis is not aligned with the light vector.

## 4. Chromalveolates

Chromalveolates (Cavalier-Smith 1999*b*) comprise the chromists (heterokonts, haptophytes, cryptophytes) and the alveolates (dinoflagellates, apicomplexans and ciliates). Chromists are a group of secondary algae that harbour plastids originating from a eukaryotic red alga (Cavalier-Smith 2002*a*; Yoon *et al.* 2002*b*). Some alveolates also have plastids, including many dinoflagellates or a marine relative of apicomplexan parasites (Moore *et al.* 2008). This is consistent with the idea that chromalveolates were ancestrally photosynthetic and the red algal symbiont was lost (or replaced via tertiary endosymbiosis; Yoon *et al.* 2002*a*) independently in many lineages (Cavalier-Smith 1999*b*, 2009; Reyes-Prieto *et al.* 2008). In agreement with a phototrophic, pelagic ancestry of the whole group (Cavalier-Smith 2009), phototaxis is widespread among chromalveolates, but is not restricted to photosynthetic species (e.g. Amon & Perkins 1968). However, in contrast to the likely single origin of chromalveolate plastids, phototaxis in chromalveolates originated at least three times independently (in heterokonts, in ciliates and in cryptophyte algae), in all cases employing unrelated photopigments.

All three major groups of chromists (heterokonts, haptophytes, cryptophytes) have several phototactic members. In heterokont algae, e.g. the brown phaeophyte algae *Pseudochorda* (Kawai *et al.* 1991)*, Ectocarpus* (Kawai *et al.* 1990) and *Fucus* (Robbins 1916), or the golden chrysophyte algae *Ochromonas* (Häder *et al.* 1981) and *Chromulina*, the ciliated zoospores or gametes (the ‘swarmers’) often show positive or negative phototaxis (Kawai 1992). Heterokont algae can be unicellular or form large multi-cellular bodies as the brown algae (kelps). The swarmers harbour two asymmetric cilia (a typical character of heterokonts, also called stramenopils), which are positioned laterally. One cilium directs anteriorly and carries mastigonemes, lateral stiff projections, which increase the tangential drag relative to the normal drag of the cilia so that the direction of the organism is reversed (the modified cilia pull the cell through the water). The other cilium directs posteriorly, is smooth and often shows green autofluorescence (figure 2). The autofluorescence of the posterior cilium is strongly correlated with the phototactic ability of the swarmers (Müller *et al.* 1987; Kawai 1988, 1992). In phototactic species the posterior cilium also has a swelling at its base, which is also strongly fluorescent, and is flanked by the stigma and the chloroplast (Kawai 1992). The stigma in heterokonts is most often part of the chloroplast, with the exception of Eustigmatophyceae where the stigma is cytoplasmic (Hibberd & Leedale 1972) and consists of carotenoid-containing globules. Non-photosynthetic heterokonts can also be phototactic (e.g. *Labyrinthula* sp. (Amon & Perkins 1968) or *Ulkenia* sp. (Amon & French 2004)). In these species, the stigma is formed in the cytoplasm by a few orange spheres. In heterokonts, the anterior hairy cilium is used for swimming, and the posterior smooth one for steering as it can bend abruptly upon stimulation (Geller & Muller 1981). This distinction of swimming and steering cilia is also present in dinoflagellates (Hand & Schmidt 1975), and reoccurs in animal larvae (see below). Heterokonts also swim in a spiral, and this spiralling is essential for phototaxis (e.g. Kawai *et al.* 1990, 1991). Spectroscopic measurements indicated that the green fluorescence in phototactic heterokonts is due to a flavin-like substance (Kawai 1988), which most likely acts as the photoreceptor during phototaxis, given the action spectrum of the behaviour (Kawai *et al.* 1990, 1991). A fluorescent flavoprotein, which was bound non-covalently to flavin mononucleotide, has recently been purified from the posterior cilium of the brown alga *Scytosiphon lomentaria*. This 41 kDa protein is related to Old Yellow Enzymes (Fujita *et al.* 2005), a family of NADH:flavin oxidoreductase/NADH oxidases with a TIM-barrel fold. These enzymes are mostly found in bacteria and fungi. The brown algal sequence is closely related to cyanobacterial ones (52% identity) and could have originated from the red algal symbiont. Whether this protein is a bona fide phototaxis photoreceptor, and if yes, how it signals to the posterior cilium, requires further studies. The posterior cilium of heterokonts also contains a pterin-like pigment that may also have a role in phototaxis (Kawai *et al.* 1996).

Much less is known about photic behaviour in haptophytes, the second chromist group. Stigmata have been described in a few species (*Diacronema* and *Pavlova* sp.; Green 1980), but phototaxis has not been observed in *Pavlova* (Foster & Smyth 1980). *Exanthemachrysis gayratiae* has a stigma and is phototactic (Gayral & Fresnel 1979), while *Coccolithus huxleyi* is phototactic but has no stigma (Mjaaland 1956). Nothing is known about photopigments in this group.

Cryptomonad algae, belonging to the third group (cryptophytes) of chromists, can also show either positive or negative phototaxis (e.g. *Cryptomonas* sp., *Chroomonas* sp.; Häder *et al.* 1987; Erata *et al.* 1995). A stigma can be present in cryptomonads. It consists of one layer of globules located in the middle of the cell, inside the chloroplast (Dodge 1969; Lucas 1982). The stigma forms an out-bulging of the chloroplast and is covered by four membranes (the rough endoplasmic reticulum (ER) of the host, the ex-plasma membrane of the red algal symbiont and the double plastid membrane of the cyanobacterium-derived chloroplast of the red alga; figure 2). Even though the plastids share common red-algal ancestry with those of other chromists, cryptomonads use retinal and SR, and not a flavin-based photopigment for phototaxis (Sineshchekov *et al.* 2005).

The SR of the cryptomonad *Guillardia theta* does not align well with green algal rhodopsins (Sharma *et al.* 2006). It also lacks the extra C-terminal domain, and may represent an independent horizontal gene transfer event from a prokaryote. Regardless of the history of the green algal and cryptomonad rhodopsins, the independent origin of phototaxis in green algae and cryptomonads is indicated by the non-homology of their stigmata. Green algae form a stigma in the cyanobacterium-derived plastid, and cryptomonads in a red alga-derived plastid.

Phototaxis is also present in several alveolates (in dinoflagellates and ciliates). Dinoflagellates can be phototactic and can have simple stigmata (e.g. *Peridinium* (Messer & Benshaul 1969), *Glenodinium foliaceum* (Dodge & Crawford 1969)). Stigmata are present in approximately 5 per cent of the species, which are predominantly freshwater ones. However, many dinoflagellates are phototactic even in the absence of a stigma (e.g. Hand & Schmidt 1975). In these species, the cell body and plastids provide the shading function. The stigma, when present, shows remarkable structural variety in dinoflagellates (Dodge 1983). It can consist of simple cytoplasmic carotenoid-containing droplets (Dodge 1983), can be part of a vestigial, three membrane-covered plastid (Dodge & Crawford 1969) or a diatom-derived real plastid (Messer & Benshaul 1969), which originated via tertiary endosymbiosis (Bhattacharya *et al.* 2004). Some species even have a light-focusing lens in association with the stigma and a putative photosensory ‘retinoid’ (Francis 1967). In dinoflagellates, one of the two cilia lies in a transverse groove (cingulum), the other one in a longitudinal groove (sulcus), which form between the thecal plates (figure 2). The stigma is always located posteriorly, underneath the groove of the longitudinal cilium. The stigma can be associated with a ‘lamellar body’, a spectacular membranous organelle with closely stacked, ER-derived flat vesicles, reminiscent of membrane stacks in animal photoreceptors (Dodge & Crawford 1969). In dinoflagellates, during axial rotation the phototransductory cascade triggers the lateral movement of the posterior, longitudinal cilium (the steering cilium) (Hand & Schmidt 1975). The photopigment of dinoflagellate phototaxis is not known, but the best candidate is a type I rhodopsin, which has been identified in the dinoflagellate *Pyrocystis lunula*. This dinoflagellate type I rhodopsin may share common ancestry with cryptomonad rhodopsins (Ruiz-Gonzalez & Marin 2004).

Many ciliates (e.g. *Ophryoglena flava* (Cadetti *et al.* 2000), *Stentor coeruleus* (Song *et al.* 1980)*, Chlamydodon mnemosyne* (Kuhlmann & Hemmersbach-Krause 1993)) are also able to perform three-dimensional phototaxis (for an overview, see Kuhlmann 1998). Ciliates show a large variety of cell biological solutions (Kuhlmann 1998), and clearly evolved phototaxis independently from other chromalveolates. *Chlamydodon mnemosyne* shows negative and positive phototaxis, depending on the feeding status of the cell. Under-fed cells are positively phototactic and form a stigma composed of several hundred orange vesicles, which accumulate at the anterior end of the cell (Kuhlmann & Hemmersbach-Krause 1993). The plasma membrane overlying the stigma contains a tightly localized autofluorescent substance, which is most likely the photoreceptor (Selbach & Kuhlmann 1999). Well-fed cells lose the stigma but retain the localized photoreceptor, and become negatively phototactic. In this case, the shading function is probably provided by the food vacuole (Selbach & Kuhlmann 1999). Several histophagous ciliates of the order Hymenostomatida are phototactic and contain a watch-glass organelle, also called Lieberkühn's organelle. This is a curved, refractive body located in the oral cavity of the cell. In *Ophryoglena* sp., the removal of the watch-glass organelle results in a loss of phototaxis consistent with a role in the detection of light direction (Kuhlmann 1998). In these ciliates, the sign of phototaxis also depends on the state of cell starvation.

Other ciliates lack conspicuous stigmata but can be phototactic. In *S. coeruleus* the cell surface bears a series of longitudinal bands with alternating clear and pigmented stripes. The pigment comes from small, pigment-containing vesicles distributed longitudinally along the cell body between the ciliary rows (Huang & Pitelka 1973). In *Stentor* and the related heterotrich ciliate, *Blepharisma japonicum*, hypericin-like molecules (called stentorin (Tao *et al.* 1993) and blepharismin (Checcucci *et al.* 1997), respectively) serve as the photoreceptor pigment (Wood 1976). The pigment is bound to protein and may trigger changes in ciliary beating by proton release into the cytoplasm (Walker *et al.* 1979). The deployment of these hypericin-like photopigments in rows of vesicles in heterotrich ciliates represents another independent origin of phototaxis.

The mechanism of steering in ciliates is unknown, but it is conceivable that in heterotrich ciliates each vesicle and the associated cilia form an independent miniature stigma and phototactic steering device. As the cell rotates, different rows of pigment vesicles and cilia will be exposed to light and react to it, each contributing to phototactic steering.

## 5. Excavates

Excavates is a diverse group of ancestrally biciliate protists, often with a ventral feeding groove. Phagotrophic, parasitic (e.g. *Giardia*) and photosynthetic species are all present. Some euglenozoa have a plastid, which originated via secondary endosymbiosis from green algal prey. These phototrophic species (e.g. *Euglena gracilis*, *Phacus pleuronectes, Trachelomonas* sp.) are also phototactic and have a red carotenoid-based shading stigma and a photosensory swelling (paraflagellar body) at the base of the long, hairy cilium.

Phototaxis is best understood in *E. gracilis*. Contrary to green algae and most chromists, the stigma in *Euglena* is not part of the chloroplast. It is found in the cytoplasm, close to the base of the cilia and is formed by membrane-bound lipid droplets that contain carotenoid pigment. The presence of both the stigma and the adjacent paraflagellar body is required for normal phototaxis. Interestingly, in *Euglena* the direction sensing is brought about by a dichroic mechanism (the paraflagellar body has a paracrystalline structure). Stigma-less mutants are phototactic, but swim in two directions. In wild-type cells the stigma blocks one of the preferred directions (Häder 1987).

The paraflagellar body contains a flavin-binding photoreceptor. In *Euglena* it has been identified as photoactivated adenylyl cyclase (PAC). PAC mediates both positive and negative phototaxis (Ntefidou *et al.* 2003), as well as a step-up photophobic response (Iseki *et al.* 2002). PAC is a heterotetrameric blue-light photoreceptor consisting of two closely related subunits, PACα and PACβ. Both subunits contain two flavin-binding BLUF (sensors of blue light using FAD) domains and two class III adenylyl cyclase (AC) domains. The adenylyl cyclase activity of the protein is elevated up to 80-fold under blue light (Iseki *et al.* 2002).

PAC is present in phototrophic euglenoids and also in kinetoplastid trypanosomes, a group of parasitic, non-phototactic excavates. The BLUF and AC domains in euglenoids are clearly related to bacterial proteins. Some bacteria have BLUF and AC domains in one protein (e.g. the gammaproteobacterial *Beggiatoa* sp. ZP_01999737), but in eukaryotes it is not found outside Euglenozoa. PAC therefore probably originated via horizontal gene transfer from bacteria and was recruited as a photoreceptor regulating photobehaviour in Euglenozoa. Whether the green algal plastid originated early or late in euglenid evolution is debatable (Cavalier-Smith 1999*a*; Leander 2004). The presence of PAC in trypanosomes may suggest that these organisms also had phototrophic and phototactic ancestors.

One euglenoid, *Peranema trichophorum*, uses a rhodopsin photopigment to control the probability of its curling behaviour. However, *Peranema* lacks a stigma and is not capable of true phototactic orientation (Saranak & Foster 2005).

## 6. Rhizaria and apusozoa

Rhizaria are mostly amoeboid unicellular protists with fine filose or reticulated pseudopodia (Cavalier-Smith 2002*b*; Nikolaev *et al.* 2004). The major groups of Rhizaria are Radiolaria, Foraminifera and Cercozoa. They often build shells from various materials.

The only photosynthetic group within Rhizaria are the chlorarachnean algae, biflagellate amoebae (Moestrup & Sengco 2001), which evolved when a cercozoan acquired a plastid of green algal origin. Similar to cryptomonads, the eukaryotic algal symbiont retained a miniature nucleus, the nucleomorph. In agreement with the amoeboid nature of chlorarachneans, they lack stigmata and three-dimensional phototaxis (Moestrup & Sengco 2001).

Foraminifera are predominantly marine with reticulated, anastomosing pseudopods and organic or calcareous tests. They can be both benthic and planktonic. Some large species harbour symbiotic algae and can show photoresponses. As foraminiferans use pseudopods for movement and lack spiral ciliary swimming, they are unable to perform three-dimensional phototaxis. A slow crawling positive phototactic reaction was described for *Amphistegina radiata* (Zmiri *et al.* 1974).

Apusozoa is a protist group of uncertain phylogenetic position (Moreira *et al.* 2007). Its members are biciliate and have a posterior cilium, which is used for gliding over surfaces (Cavalier-Smith 2009). No phototaxis or stigmata are present in these benthic gliders.

## 7. Amoebozoa

Amoebozoa comprise solitary and social amoebae. Even though they can have cilia, three-dimensional phototaxis is only known from the aciliate soil-dwelling social amoeba, *Dictyostelium discoideum*. Upon starvation, individual amoeboid cells aggregate and form polarized, multi-cellular slugs, which are organized by cAMP signalling. The whole slug moves in the soil by axial rotation and migrates to the surface to form fruiting bodies using positive phototaxis (Francis 1964; Fisher 1997; Miura & Siegert 2000). *Dictyostelium* is the only organism known to date that can perform helical three-dimensional phototactic navigation without using cilia. The strategy of *Dictyostelium* phototaxis is reminiscent of the three-dimensional phototaxis of pelagic species. As the slug rotates, its anterior tip can turn towards the light. There is no stigma or any shading device, but the whole slug serves as a refractive lens that focuses light on the opposite side of the body (Francis 1964). This focused light then triggers the turning of the tip towards the light (Miura & Siegert 2000). A *Dictyostelium* slug is of course not able to swim in open water using amoeboid collective cell migration. However, three-dimensional orientation along the light vector is possible, provided that the slug is migrating in a dense medium (e.g. oil). The photoreceptor that mediates phototaxis in *Dictyostelium* is not known. No rhodopsin has been identified in the *Dictyostelium* genome, although several classes of G-protein-coupled receptors (GPCRs) are present (Eichinger *et al.* 2005).

A slow two-dimensional positive and negative phototactic movement has also been described for the single-celled amoebae of *Dictyostelium*, migrating on a plate (Häder & Vollertsen 1991). The amoebae use different photoreceptors than the slugs, and the mechanism of phototaxis is also different. Individual amoeba can react to local illumination either by the formation of pseudopodia at the irradiated parts (low illuminance) or the suppression of pseudopodia formation (high illuminance) (Häder *et al.* 1983).

## 8. Opisthokonts

Fungi, together with animals and related protozoan taxa (e.g. choanoflagellates, ichthyosporeans, nucleariids; Steenkamp *et al.* 2006) comprise the opisthokonts. Phototaxis is present in some chytrid fungi and is widespread in animal ciliated larvae.

The ciliated zoospores of some marine and soil chytrid fungi show phototactic responses, including *Rhizidium vorax* (Strasburger 1878), *Phlyctochytrium* sp. (Kazama 1972), *Allomyces* sp. (Robertson 1972) and *Rhizophydium littoreum* (Muehlstein *et al.* 1987). Fungi evolved a chitin cell wall early during their evolution, and lost cilia at least four times independently (James *et al.* 2006). Only ciliated chytrid fungi are able to perform phototaxis. Phototactic chytrids lack well-developed stigmata, but harbour large reddish vesicles near the base of the single posterior cilium, which form a ‘side-body complex’ (Robertson 1972; Saranak & Foster 1997). These vesicles can provide the shading function. The photoreceptor in *Allomyces* was shown to be a rhodopsin that is localized in the plasma membrane (Saranak & Foster 1997). No rhodopsin sequence has yet been reported from *Allomyces*, but the action spectrum suggests a type II rhodopsin (Saranak & Foster 1997). Other fungi have type I rhodopsins (Bieszke *et al.* 1999; Idnurm & Howlett 2001), which were acquired via horizontal gene transfer from prokaryotes, independent of algal rhodopsins (Sharma *et al.* 2006). The chytrid phototactic pigment awaits molecular characterization. No phototaxis has been reported in Choanozoa (the basal taxon comprising ancestral opisthokont protists), including choanoflagellates, nucleariids and ichthyosporeans. These organisms are either amoeboid without ciliated stages (nucleariids) or are primarily benthic and use their single cilium together with an actin-based collar for feeding (choanoflagellates). The lack of cilia and the amoeboid and benthic nature of these organisms are consistent with a general lack of stigmata and phototaxis. Choanoflagellates are the sister protist group to animals (King 2004). Animals ancestrally were therefore most likely benthic, but evolved pelagic larval stages very early on (Nielsen 2008). Ciliated pelagic larvae are widespread in animals. They are present in sponges, cnidarians and many bilaterian invertebrates (Young 2002). Ciliated animal larvae always show helical swimming and very often are phototactic. Phototaxis is present in the ciliated planula larvae of demosponges and promotes the dispersal of the larvae (Leys & Degnan 2001). The larvae of demosponges, such as *Amphimedon queenslandica*, are propelled by short (20 µm) motile cilia, which cover almost the whole larva and emanate from columnar epithelial cells. At the posterior end of the larva, there is a ring of specialized photoreceptor cells that regulates phototactic steering. These cells have photosensory membranes, contain shading pigment granules and also carry a long cilium (120–150 µm), which steer the larva by bending upon directional light stimuli (Leys & Degnan 2001). Thus, in *Amphimedon* larvae, the photosensory, shading and steering functions are combined in one cell. These sponge larvae have clear anteroposterior (A-P) patterning but no dorsoventral (D-V) patterning (Adamska *et al.* 2007). As the radially symmetrical larva swims and rotates under lateral illumination, a given segment of the photosensory ring is turned towards the light, bends its cilia and steers the larva. Steering continues until the larva is aligned with the light vector and illumination is uniform.

In phototactic cnidarian larvae, the photosensory, shading and steering functions can also be combined in one cell. In the multi-functional photoreceptors of the larvae of the box jellyfish *Tripedalia*, a steering cilium presumably also changes its bending angle upon illumination (Nordström *et al.* 2003).

Bilaterian ciliated larvae broke up the radial symmetry by D-V patterning and often have a pair of bilateral eyespots, consisting of distinct photoreceptor and pigment cells (the term eyespot in animal larvae refers to the organ). In the simplest case, the eyespot consists only of two cells, a photoreceptor and a shading pigment cell, as in the ciliated larvae of the annelid polychaete *Platynereis dumerilii*. In *Platynereis*, the eyespots have a wide, conical, laterally directed field of view (Jékely *et al.* 2008). Phototaxis is regulated via neuronal contact between the eyespot and the ciliary band, which propels the larva. The eyespot photoreceptor is a cholinergic neuron, which directly innervates the ciliary band. Upon light exposure, cholinergic neurotransmission by the photoreceptor slows down the beating of adjacent cilia of the ciliary band, resulting in the reorientation of the helical trajectory towards the light. As the larva rotates around its A-P axis, it can steer twice during a full axial rotation. If one eyespot is surgically removed, the larva is still phototactic, but can now only steer once per full rotation (Jékely *et al.* 2008), analogous to the situation in protists with one stigma (Foster 2009). Other ciliated bilaterian larvae probably use a similar mechanism and directly regulate ciliary beating during phototaxis by the eyespots. Phototaxis and simple cellular eyespots have been described in the larvae of many marine species, including bryozoans (Pires & Woollacott 1997), polychaetes (Marsden 1984), nemertines (Smith 1935) and hemichordates (Brandenburger *et al.* 1973); for an overview, see Thorson (1964).

Animal photoreceptors use type II rhodopsins to detect light. Whereas type I rhodopsins function as either light-driven ion pumps or light-gated ion channels, or interact with various transducer proteins, type II rhodopsins are members of the superfamily of GPCRs and signal via heterotrimeric G-proteins (Spudich *et al.* 2000). Rhodopsins are present in most animals (one notable exception is *Caenorhabditis elegans*; Bargmann 1998) and trace back at least to the cnidarian–bilaterian last common ancestor. In sponges, no rhodopsin gene has yet been identified (Plachetzki *et al.* 2007). In cnidarians, there are several rhodopsins (Plachetzki *et al.* 2007; Kozmik *et al.* 2008; Suga *et al.* 2008), although none has yet been shown to regulate phototaxis. In *Platynereis* larvae, the eyespot photoreceptor expresses a new rhabdomeric-type rhodopsin (G. Jékely 2009, unpublished data).

It has often been suggested that animal type II rhodopsins may have evolved from microbial type I rhodopsins, given the same 7-transmembrane topology, the conserved lysine in the seventh transmembrane segment and the binding to retinal chromophore. This now seems less likely because non-opsin GPCRs clearly trace back to the unikont last common ancestor (Eichinger *et al.* 2005) and even to the eukaryotic last common ancestor (Fredriksson & Schiöth 2005). Animal type II rhodopsins are clearly more closely related to these sequences than to type I rhodopsins. The first animal rhodopsin only appears in cnidarians (Plachetzki *et al.* 2007). Rhodopsins are also absent from the choanoflagellate *Monosiga brevicollis*. This rather indicates that animal rhodopsins evolved from non-opsin GPCRs and the lysine residue in the seventh transmembrane segment, as well as the use of retinal as a chromophore in both type I rhodopsins and animal rhodopsins, is an example of molecular convergence. Further taxon sampling (e.g. chytrid and sponge genome sequences) will help to clarify the history of animal rhodopsins.

## 9. Advantages of phototaxis

Phototaxis can have several advantages for the organism. This includes the regulation of light exposure of photosynthetic algae, the finding of phototrophic organisms for food, the facilitation of larval dispersal or the increased likelihood of gamete fusion on the surface.

The first obvious advantage of phototaxis is for photosynthetic organisms that harvest light energy. This is why many planktonic algae are phototactic. These organisms have to find the optimum illumination conditions depending on the state of the electron transport chain and the time of the day (Burns & Rosa 1980). The problem is confounded by the fact that unregulated positive phototaxis to the surface layers is dangerous because it exposes the organisms to damaging UV radiation. Phototaxis therefore has to be tightly controlled. In many algae and other organisms, the sign of phototaxis depends on the intensity of light so that low intensities elicit a positive response, and high intensities a negative one (e.g. *Chlamydomonas* (Feinleib & Curry 1971), *Ochromonas*, *Euglena* (Häder *et al.* 1981)). This switch allows the selection of optimum illumination, as the radiance level changes throughout the day. The sign of phototaxis can also be modulated by photosynthetic activity, as in *Chlamydomonas* (Takahashi & Watanabe 1993).

Phototaxis can also serve to bring motile propagules of chytrid fungi towards the zones where they can potentially contact host algae (e.g. the estuary chytrid *Phlyctochytrium* sp. parasitizing the green alga *Bryopsis plumosa* (Kazama & Schornstein 1977; Muehlstein *et al.* 1987)). Eduard Strasburger observed already in 1878 that some chytrids gather in the same place as the phototactic green algae that they parasitize (cited in Saranak & Foster 1997). Another example of finding food with light is the non-photosynthetic heterokont protist, *Ulkenia* sp. *Ulkenia* shows positive phototaxis with a peak at 480 nm, which seems to be optimized to detect bioluminescence generated by its prey, *Vibrio fischeri*, living on decaying fish (Amon & French 2004).

Some organisms regulate phototaxis depending on the nutritional state. The ciliate *Chlamydodon* sp. shows positive phototaxis when is under-fed and shows negative phototaxis when well-fed. *Chlamydodon* thus minimizes the exposure to light and only swims towards the surface when feeding on phototrophic prey.

The larvae of several marine invertebrates can be positively phototactic in the non-feeding stages. The phototactic upward swimming is thought to enhance the dispersal of the larvae. The behaviour only lasts for a few days, after which several species turn negatively phototactic and settle on the substrate (Thorson 1964).

Another potential advantage of phototaxis is to increase the probability of gamete encounters. If positively phototactic gametes reach the surface, they will have a higher chance of finding mates in two dimensions (Togashi & Cox 2004). Both male and female gametes of the marine green alga *Monostroma angicava* are positively phototactic, and this behaviour was shown to increase the rate of gametic encounters (Togashi *et al.* 1999). After fertilization, the zygotes turn immediately negatively phototactic, to minimize light exposure.

## 10. How can phototaxis evolve?

All phototactic eukaryotes that are able to orient along a light vector in three dimensions use the same general strategy. Eukaryotes evolved such phototactic capacity at least eight times independently (table 1). The multiple independent origins of phototaxis in various eukaryotic groups suggest that it is not too difficult to evolve this behaviour. Our detailed understanding of phototactic navigation allows us to define the necessary cellular and behavioural features and to suggest a plausible order in which these evolved.

**Table 1.** Summary of photopigments and stigma/eyespot structures in phototactic eukaryotes.

	photopigment	stigma/eyespot	independent origin?
green algae	type I rhodopsin with large C-terminal extension, probably of independent origin from cryptophyte rhodopsin	in the cyanobacterium-derived chloroplast	yes
heterokonts	flavoprotein, pterin	in the red alga-derived chloroplast or in the cytoplasm	yes
haptophytes	?	in the red alga-derived chloroplast	?
cryptophytes	type I rhodopsin, probably of independent origin from green algal rhodopsin	in the red alga-derived chloroplast	yes
ciliates	hypericin-like pigment+protein	formed by cytoplasmic vesicles	yes
dinoflagellates	(rhodopsin ?)	none, or in the cytoplasm, or in a diatom-derived, or vestigial chloroplast	?
euglenoids	light-activated adenylyl cyclase (PAC)	formed by vesicles close to the base of the cilia	yes
Amoebozoa	? (not a rhodopsin)	none, direction sensing by lens effect	yes
chytrid fungi	type II rhodopsin (based on spectrum), origin unclear	formed by large cytoplasmic vesicle	yes
animals	type II rhodopsin (sponges may be an exception), independent origin from type I rhodopsins	pigment vesicles in the photoreceptor cell or a distinct pigment cell	yes

Question marks indicate uncertainties.

The necessary, hence universal features of pelagic, three-dimensional phototactic organisms are the following: (i) polarity and a fixed shape; (ii) spiral swimming with cilia; (iii) photosensory molecules and a phototransductory cascade that affects ciliary beating; and (iv) a shading or refractive body that ensures the orientation-dependent illumination of the photopigments during axial rotation. Given these components, we can describe the elementary steps through which phototaxis probably has evolved in most cases.

(i) *A polarized body with a fixed shape*. This evolved many times independently as previously amoeboid or sessile benthic organisms conquered the open waters (Cavalier-Smith 2009). A cell with a fixed shape and one or two cilia is intrinsically polarized with two main axes, an A-P and a D-V axis. The position of the basal bodies (one or two in unikonts, two in bikonts) and the microtubule cytoskeleton defines the A-P axis. The D-V axis is defined by the asymmetry of the ciliary root, which anchors the basal body. A stable cell shape can be maintained either by submembrane cytoskeletal elements (e.g. alveolates) or an external cell wall (fungi, plants).

(ii) *Spiral swimming*. This is a consequence of (i) and is the rule for pelagic, self-propelled ciliary swimmers with constant propulsion forces and asymmetry. All ciliated organisms that swim do so in a spiral (Jennings 1901). The spiral results from the repetition of the same elementary rotation and translation movements. During spiralling, the body rotates on its longitudinal axis and a given side is continuously directed outwards. Gliding cells that lack cilia can also perform phototaxis, such as some red algae (Nultsch & Schuchart 1980), individual *Dictyostelium* amoebae or *Euglena mutabilis* (Häder & Melkonian 1983), but these cells do not rotate, and orientation is always on a surface, in two dimensions.

(iii) *Photopigments* (the order of the origin of (i)–(iii) is not important). Photopigments often came from bacterial food via horizontal gene transfer or from the chloroplast via endosymbiotic gene transfer (animal rhodopsins are one exception). The first function of these photopigments could have been the regulation of a photophobic response, and not phototaxis. This is easier to evolve and only requires the integration of photoreceptor signalling into ciliary signalling to turn off ciliary beating. Such photophobic behaviour still coexists in many phototactic organisms and functions independent of the stigma. It also does not require the enrichment of photoreceptors in the region of the stigma.

The integration of horizontally acquired photoreceptors into pre-existing cellular signalling could have been easy for both bacteriorhodopsin and light-activated adenylyl cyclase. Bacteriorhodopsin is an autonomous light-driven ion transporter, which immediately after its acquisition could provide meaningful signals to the previously blind organisms. This may have occurred several times independently. Similarly, PAC, the light-activated adenylyl cyclase of *Euglena*, is an autonomous sensor and signal transducer that could be directly integrated into cAMP signalling cascades.

(iv) *Stigma*. Stigmata evolved next, for direction sensing and increased contrast modulation. A shading or refractive body in the cell, positioned asymmetrically and in a fixed position relative to the plane of cilia, will result in the periodic illumination of the photopigments in one part of the cell and trigger periodic signalling during axial rotation. The intensity of the signal will depend on the orientation of the body relative to the light vector. The shading function can initially be provided in a crude way by the plastid or a membrane vesicle. An example to illustrate the sometimes rather casual nature of the shading body is the ciliate *Chlamydodon*, where, following phagocytosis, the food vacuole becomes the shading organelle (Selbach & Kuhlmann 1999). The shading bodies of phototactic chytrid fungi are also very simple, consisting only of a few laterally positioned reddish vesicles (Robertson 1972; Saranak & Foster 1997). Stigmata evolved in parallel with the local accumulation of the photoreceptors. Photoreceptor enrichment next to the stigma is universal, and also increases sensitivity and the ability to detect contrast during helical swimming. A stigma is not always necessary. Many phototactic organisms can do without it and use the cell body for shading or refraction (e.g. many dinoflagellates). However, stigmata evolved when increased contrast modulation was an advantage or was necessary to evolve phototaxis at all. Enhancing contrast can, for example, be more important in turbid waters, such as lakes. In agreement with this, most dinoflagellates that have stigmata are freshwater species.

If an organism puts together components (i)–(iv), even in a crude way, it will become phototactic. The important point is that phototaxis does not necessarily require a sophisticated regulation of ciliary beating, at least during the initial stages of its evolution. It is sufficient if the periodic light stimulus triggers *some* change in ciliary beating. This will change the flow around the swimming body and change the direction of the helical trajectory. This elementary turning at every instance of light exposure continues as long as illumination is not uniform. Depending on the nature of ciliary regulation and the view angle of the photosensor, this will lead to an orientation either towards or away from the light. It is important to stress that as soon as a shading body with respect to a photosensitive patch in the membrane is placed in a D-V polarized fashion (i.e. roughly perpendicular to the axis of the swimming A-P axis) and the phototransduction triggers a change in ciliary beating, orientation will follow. As long as the D-V polarized visual axis of the cell is not perpendicular to the light vector, periodic signalling and a periodic readjustment of the helical trajectory will happen. When the cell is oriented along the light vector, there will be no intensity changes during axial rotation, hence no differential signalling and no turning.

A crude form of phototaxis can be optimized for many parameters. Sensitivity can increase by improving the absorptive/reflective power of the stigma (Kreimer 1999), by the concentration of the photopigment or by the evolution of signal amplification (Sineshchekov *et al.* 2009). Mechanisms to switch from positive to negative phototaxis beyond an intensity threshold can evolve to minimize UV exposure. This can, for example, evolve by introducing a delay in the phototransductory cascade. If the signal is delayed with the time that corresponds to half axial rotation, the sign of phototaxis will reverse (K. W. Foster 2009, personal communication).

## 11. Phototaxis and benthic–pelagic transitions during eukaryote evolution

Recent advances in our understanding of eukaryote phylogeny and the rooting of the eukaryote tree allow a more reliable reconstruction of the last common eukaryote ancestor (Stechmann & Cavalier-Smith 2002; Cavalier-Smith 2003, 2004; Keeling *et al.* 2005; Baldauf 2008). It seems now likely that the last common eukaryote ancestor was a benthic amoeboflagellate with one or, less likely, two cilia, and the ability to form pseudopods (Richards & Cavalier-Smith 2005; Cavalier-Smith 2009). In an amoeboflagellate cell, the cilium is usually not used for swimming, but for collecting food particles via undulatory motion and ciliary surface motility. Amoeboid movement entails the extension of pseudopods and constant shape changes. An amoeboflagellate cell, therefore, even if it had a stigma, would not be able to perform phototaxis in open water because the stigma would not have a fixed view angle and a fixed position relative to the cilium. For efficient phototaxis, the cell has to have a constant shape. A fixed cell shape evolved many times independently in eukaryotes. Plants evolved a cell wall, alveolates evolved cortical alveoli (possibly already in the plant-chromalveolate common ancestor) and subpellicular microtubules (Gould *et al.* 2008). Excavates evolved rigidifying pellicle strips, composed of articulins (Huttenlauch & Stick 2003), which run underneath the plasma membrane from anterior to posterior (Leander & Farmer 2000). Fungi evolved chitin cell walls, and animals evolved multi-cellular tissues that are held together by cell adhesion and organized by developmental signalling and planar polarity. All of these groups probably ancestrally lack pseudopodia and amoeboid motility, and only occasionally re-evolved them. In the common ancestor of plants and chromalveolates, as well as in excavates, the loss of amoeboid motility and the evolution of a fixed cell form probably happened in parallel with the transition from a benthic to a pelagic lifestyle (Cavalier-Smith 2009). Likewise, in animals, the first polarized tissues evolved in the planktonic larval stages of sponges (Nielsen 2008). The origin of phototaxis recurrently followed the origin of pelagic forms (figure 2). Organismal rigidity was a prerequisite for the evolution of phototaxis. When phototaxis appeared, it had obvious advantages for the pelagic organisms.

## 12. Conclusions

Phototaxis, which allows orientation along a light vector in three dimensions, is unknown in prokaryotes. In contrast, in eukaryotes, it evolved at least eight times independently (table 1). Phototaxis appeared in these lineages after they evolved a planktonic lifestyle with ciliary swimming (with the exception of *Dictyostelium*) and a fixed shape. The photopigments were often acquired via horizontal gene transfer from a prokaryotic source. The signals generated by these autonomous photopigments (ion currents or changes in cyclic nucleotide levels) could be integrated relatively easily into eukaryotic ciliary signalling. The photopigments could first have mediated a general photophobic response that evolved into phototaxis when the cells developed shading stigmata and concentrated the photopigments in their vicinity. These simple elementary steps, all possibly with an adaptive significance, explain why phototaxis could evolve so many times independently.
